# Joint Optical and Wireless Resource Allocation for Cooperative Transmission in C-RAN

**DOI:** 10.3390/s21010217

**Published:** 2020-12-31

**Authors:** Peng Yang, Liao Chen, Hong Zhang, Jing Yang, Ruyan Wang, Zhidu Li

**Affiliations:** 1 School of Communication and Information Engineering, Chongqing University of Posts and Telecommunications, Chongqing 400065, China; yangpeng@caict.ac.cn (P.Y.); s180101046@stu.cqupt.edu.cn (L.C.); yangjingb@cqupt.edu.cn (J.Y.); wangry@cqupt.edu.cn (R.W.); lizd@cqupt.edu.cn (Z.L.); 2 Chongqing Key Laboratory of Optical Communication and Networks, Chongqing 400065, China; 3 Chongqing Key Laboratory of Ubiquitous Sensing and Networking, Chongqing 400065, China; 4 West Institute of CAICT of MIIT, Chongqing 400065, China

**Keywords:** C-RAN, TWDM-PON, cooperative transmission, resource schedule

## Abstract

Cooperative multipoint transmission (CoMP) is one of the most promising paradigms for mitigating interference in cloud radio access networks (C-RAN). It allows multiple remote radio units (RRUs) to transmit the same data flow to a user to further improve the signal quality. However, CoMP may incur redundant data transmission over fronthaul network in the C-RAN. In a C-RAN employing CoMP, a key problem is how to coordinate heterogeneous resource allocation to maximize the cooperation gain while reducing the fronthaul load. In this paper, the cooperation transmission based on a multi-dimensional resource schedule (MRSCT) scheme, jointly considering user association, spectrum resource allocation, and wavelength resource allocation, is firstly envisioned in the underlying C-RAN integrating time and wavelength division multiplexing passive optical network (TWDM-PON) to maximize fronthaul efficiency. Then a two-timescale resource allocation framework including two sub-approaches is established. More specially, the first sub-approach mainly focuses on exploiting reinforcement learning to obtain a wavelength resource allocation strategy to relieve fronthaul traffic load. Moreover, the second sub-approach adopts the overlapping coalition formation game to establish a user-centric cooperative set, where spectrum resources are dynamically allocated to further alleviate the interference issue. The theoretical analysis and simulation results validate the performance of MRSCT scheme on the fronthaul efficiency, user experience, and system service capability.

## 1. Introduction

With the development of the information and communications technology and the popularization of smart mobile terminals, wireless network operators are faced with the increasing demand for high-speed data traffic [1]. Besides, the emergence of new services, such as 4K high-definition video and VR promoted the transformation from traditional connection-centric to content-centric, and traffic types also shifting from telephone calls and text messages to video streaming and content sharing [2]. As a potential solution meeting the imperious needs of the next generation access network, the cloud radio access network (C-RAN) has attracted extensive research attention in the research community recently [3].

C-RAN paradigm is served as a green radio access network with the characteristics of centralized processing, cooperative radio, and real-time cloud computing [4,5]. In C-RAN, traditional base station is divided into three parts, i.e., remote radio units (RRUs), a centralized baseband unit (BBU) pool, and the BBU and RRUs are connecting by the fronthaul network [6]. Apart from the potential advantages of wider coverage and higher capacity, C-RAN also supports autonomous network management, such as self-optimization, self-adaptation, and self-configuration [7].

In C-RAN, both network function virtualization (NFV) and centralized signal processing facilitate the realization of coordinated multi-point transmission (CoMP) to alleviate the interference among users. In practice, cooperation gain is limited by the congested bandwidth of fronthaul or backhaul [8]. More specifically, the redundant data transmission caused by CoMP will further increase the burden of the fronthaul network, further reducing the performance of the C-RAN fronthaul network. In addition, the fronthaul capacity constraint has a great impact on resource scheduling for cooperative transmission [9]. Time and wavelength division multiplexing passive optical network (TWDM-PON) is a promising candidate to provide high bandwidth fronthaul network. However, optical link is still difficult to meet the large-scale traffic requirements of fronthaul network [10].

Reducing the fronthaul load has been regarded as an effective solution to improve the system service capacity. On one hand, edge cache has been proposed as an effective solution to moderate the fronthaul link pressure [11,12,13]. Moreover, the introduction of the edge cache into the C-RAN employing CoMP can ease the contradiction between the fronthaul bottleneck and the wireless side performance [14]. On the other hand, fronthaul resource re-allocation among RRUs can further balance fronthaul load [15].

Therefore, joint resources optimization in optical domain and wireless domain for CoMP emerge as a key issue in C-RAN integrated with TWDM-PON. For users suffering strong interference, RRU with requested content may not meet the user’s requirement in terms of RB. To achieve the scalability of user QoS requirements, more RRUs are needed to provide a higher signal-to-noise ratio (SINR) via consuming fewer RB. However, payload sharing causes repeated transmission of content in the different wavelengths, leading to a large amount of fronthaul resource waste.

Existing works have optimized the resource allocation problem of the wireless side and the optical side under C-RAN architecture considering the cooperative transmission. Although several works consider the heterogeneous resource allocation simultaneously, wireless backhaul and optical fronthaul are scheduled separately. In practice, the available fronthaul bandwidth of the RRU will become the bottleneck of the achievable rate of users on the wireless side. Meanwhile, the different load of fronthaul which is generated by the resource allocation strategy on the wireless side will also affect the allocation of optical resources.

To maximize the transmission efficiency in non-ideal fronthaul, we first propose a multi-dimensional resource schedule-based cooperative transmission (MRSCT) scheme under C-RAN integrated with TWDM-PON, jointly considering cache resources, wavelength resources, and wireless resources. Then, a reinforcement learning-based wavelength allocation (RLWA) algorithm is performed considering the network load and the achievable throughput according to the transmission scheme. To further improve the service capability of RRU, we finally propose CoMP clustering and dynamic spectrum allocation (CCDSA) algorithm to provide an efficient CoMP clustering according to the wavelength allocation result of RLWA algorithm. Then both user’s cooperative sets and resource block is optimized. It is demonstrated that the proposed MRSCT scheme outperforms existing transmission schemes in terms of user rate, system throughput, and resource utilization. The main contributions of this paper can be summarized as follows:

(1) In our work, TWDM-PON is introduced as fronthaul network for CoMP. A two-timescale resource allocation framework is proposed for a cooperative transmission scenario. The objective is to jointly optimize the CoMP clustering, RB allocation and fronthaul bandwidth.

(2) This work concentrates more on the joint optimization of wavelength in optical domain and cooperative set selection in wireless domain. Then an RLWA algorithm and a CCDSA algorithm are jointly designed to maximize cooperation gain while reducing the fronthaul load.

(3) The convergence and complexity of the proposed RLWA algorithm and CCDSA algorithm are analyzed. Particularly, the cluster formation result is proved to be Nash-stable. In addition, we verify the effectiveness of MRSCT scheme by simulations.

The remainder of the paper is organized as follows. We summarize and discuss related work in Section 2. System model is introduced in Section 3. The problem formulation and our proposed scheme are presented in Section 4. In Section 5, we evaluated the proposed scheme by plenty of simulations. Finally, Section 6 concludes the paper.

## 2. Related Work

Recently, some researchers have conducted corresponding research on resource allocation strategy considering CoMP from the wireless perspective [16,17,18,19,20]. For example, Chen et al. analyzed joint transmission performance through random geometry [16]. Then the network-centric solution is proposed in [17,18], in which cooperative sets and resource allocation were jointly optimized to achieve better network performance, such as network throughput and coverage probability. To improve the quality of service (QoS), Wu et al. considered a user-centric scheme to mitigate inter-cluster interference [19]. Note that cooperation gain can be significantly improved with the increase of cluster size but declines as the base station load increases [20]. To address this issue, Lai et al. constructed the cooperative sets for the user based on access point load, and vertex coloring was adopted to avoid resource conflicts among users [21].

However, the prior works are carried out under ideal fronthaul or backhaul links. Motivated by this, Ha et al. proposed two low-complexity algorithms, in which the cooperative set, precoding, and power allocation scheme are jointly designed under fronthaul constraint [22]. Similarly, multicast technology was introduced into wireless backhaul, combining multicast beamforming and RRU clustering for sum-rate maximization [23]. However, the researches mentioned above have considered resource scheduling in a fixed fronthaul link. In this paper, we adopt TWDM-PON as fronthaul network. To flexibly adapt to load requirements, the wavelengths allocated to each RRU can be adjusted dynamically.

In a C-RAN integrated with optical fronthaul, the joint optical and wireless resource allocation should be considered [24]. To enhance the ability to respond the dynamic demand of end-to-end users, joint optimization of multiple dimensional resources including radio and optical was considered in [25,26,27] for service provisioning in Cloud Radio over Fiber Network (C-RoFN). However, the above work was not carried out in a cooperative transmission scenario. Taking CoMP into consideration, the impact of the reconstruction of the optical fronthaul network on the performance of CoMP was studied in [28]. Zhang et al. reconstructed the link from RRU to BBU through wavelength allocation to improve the cooperation gain in [28]. A compromise is achieved between beamforming throughput and fronthaul bandwidth by coordinating wireless resources and optical resources in a C-RAN [29]. With the help of multicast technology, a scheme combining fronthaul bandwidth allocation, multicast beamforming, and RRU clustering was proposed to improve fairness among groups in [30]. However, the above research make decisions of fronthaul and wireless resources schedule at the same timescale, which results in frequent wavelength switching and additional overhead inevitably. Considering the periodic behavior of user traffic, it is necessary to pre-allocate wavelength resources.

## 3. System Model

**Figure 1:**
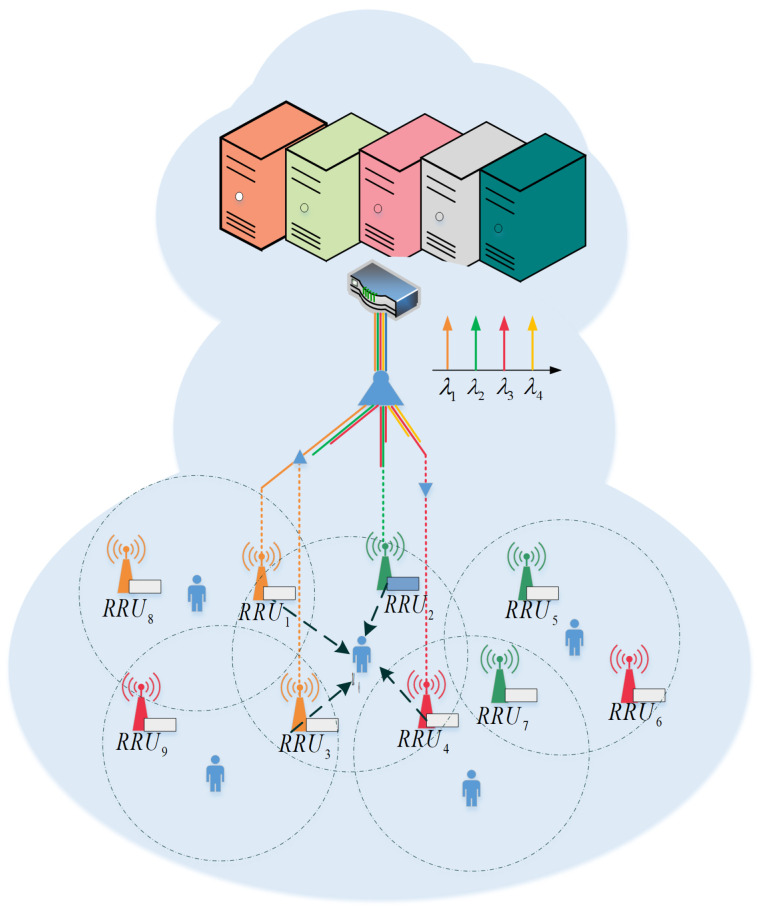
System Model.

We consider a C-RAN composed of distributed RRUs and a centralized BBU, between which is a low-latency, high-bandwidth TWDM-PON. To reduce the fronthaul load, the BBU and RRU are equipped with a cache, as shown in Figure 1. As the cache capacity of BBU is much larger than RRU, it is assumed that the cooperative hierarchical cache of BBU and RRU is sufficient to meet the traffic demand in the network. Let $F_{\Omega }=\left\{1,2,\cdots ,f,\cdots F\right\}$ denote the index of all content files available for request. We consider a resource schedule under a BBU which stored all files.

Let $M_{\Omega }=\left\{1,2,\cdots ,m,\cdots M\right\}$ denote the set of RRUs in the considered C-RAN, $K_{\Omega }=\left\{1,2,\cdots ,k,\cdots ,K\right\}$ denote the set of active users in the system. We consider a discrete-time slot system, in which users arrive and initiate a request randomly. We assume that there is a centralized controller in the BBU, and it can obtain all requests and channel state information under the BBU. Jointly determined by the user’s request, the radio resources and wavelength configuration of the RRU, users can acquire contents in three ways, as shown below: (i) When a user makes a request to RRU for content that is stored in the local edge-cache, it can be served locally. (ii) When a user makes a request to RRU for content not existing in the current RRU cache, it has to fetch the requested content file from the fronthaul. (iii) When a user is served cooperatively, to support the collaboration with the locally responding RRUs, the RRU without the requested content needs to fetch content from fronthaul.

To explore the non-uniformity of user requests in the time and space domain, we adopt TWDM-PON as fronthaul. Then wavelengths are allocated flexibly to meet the fronthaul load requirements. Moreover, the data redundancy of the fronthaul can be reduced by the broadcast characteristics of TWDM-PON. Considering RRUs with large differences in popularity distribution require larger fronthaul bandwidth, we then design a resource allocation scheme involving both optical and wireless resources to enhance RRU collaboration opportunities, improving the transmission quality while reducing the fronthaul load.

## 4. Joint Scheduling of Optical and Wireless Resources for Cooperative Transmission

### 4.1. Two-Timescale Resource Allocation Framework

The goal of this paper is to improve the efficiency of transmission, which is restricted by fronthaul bottleneck and limited spectrum resources. Early works always optimize optical and wireless resources in a single timescale framework. To adapt to the real-time changes in wireless channel, the duration of the timescale is designed to be short to meet the characteristics of wireless channels. However, wavelength allocation should be performed on a longer time scale, since the load of wavelength changes relatively slowly.

**Figure 2:**
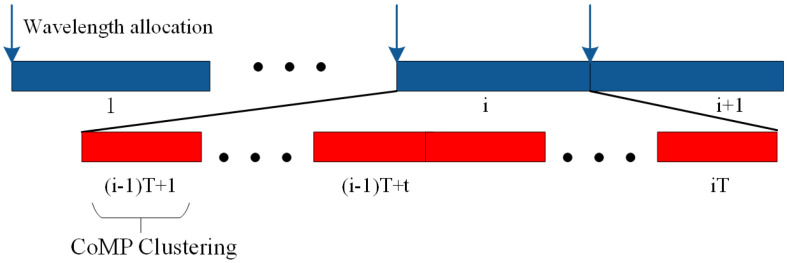
Two-timescale Resource Management.

As the wavelength resource and wireless resource allocation occur in different timescales, we establish a two-timescale resource allocation framework [31]. As shown in Figure 2, the system time is divided into multiple time frames, marked as *i*, then each RRU is assigned a wavelength based on the long-term utility at the beginning of each time frame. Each time frame contains *T* time slots in which a cooperative set is formed for the user according to the current network conditions, and then spectrum resources are allocated.

The two-timescale resource management framework can capture the stochastic nature of request distribution and channel conditions by allocating wavelengths in a longer timescale. It is assumed that the wavelength assignment matrix is fixed in each time frame, then we can derive the cooperative sets for each user in a timeslot. Let $\psi_{Y}^{*}\left(H,Q\right)$ define clustering result by the given wavelength allocation matrix *Y*, the current channel state information *Q*, and request distribution *H*. According to the three above matrix, we can assign wavelengths to RRUs at the beginning of a time frame. With wavelength allocation determined, a user-centric cluster is formed under the constraints of fronthaul capacity, effectively increasing system throughput and fronthaul efficiency.

### 4.2. Reinforcement Learning-Based Wavelength Allocation Algorithm

Since the performance of CoMP cooperative set is limited by available fronthaul bandwidth of RRUs which participating in collaboration, we propose an RLWA algorithm concerning fronthaul load caused by cooperative sets. RRUs belong to the same wavelength have more opportunities to collaborate since the data shared by cooperative RRUs are transmitted only once. In other words, RRU distributed in areas with high similarity of requested content generates less fronthaul demand. As the similarity decreases, RRUs need more fronthaul bandwidth to guarantee the access network performance when users cannot be served locally. In this section, taking the RRUs’ behavior in short timescale into account, then a wavelength allocation algorithm in long timescale according to the clustering result is designed.

To relieve the contradiction between fronthaul overhead and access layer performance, we propose an algorithm considering the gain of cluster formation in the wavelength allocation process. Let $y_{mn}$ denote the wavelength allocation indicator, whereas $y_{mn}\;=\;1$ means that wavelength *n* is allocated to RRU *m*. Otherwise $y_{mn}\;=\;0$. If the wavelength *n* is allocated to any RRUs at the beginning of a time frame, it is considered to be occupied in all time slots regardless of how much data is carried. Therefore, in our algorithm, the fronthaul load is defined as the total capacity of occupied wavelength in a time frame. Let $u(\psi_{Y}^{*}\left(H,Q\right))$ denote the throughput in each transmission slot. Then fronthaul efficiency $\eta_{\mathrm{FE}}$ is defined as the amount of data transmitted by the unit fronthaul, which can be calculated as Equation (1)

(1) $$\eta_{\mathrm{FE}}=\frac{u(\psi_{Y}^{*}\left(H,Q\right))}{\sum_{n}V_{n}D_{\max}}$$

Then in each time frame, the long-term revenue of the system can be expressed as the expected value of the fronthaul efficiency during that period, and the problem of wavelength allocation can be modeled as follows:

(2) $$\max_{Y}\frac{E_{H,Q}\left[u\left(\psi_{Y}^{*}\left(H,Q\right)\right)\right]}{\sum_{n}V_{n}D_{\max}}$$

(3) $$s.t.V_{n}={𝟙}_{\{\sum_{m\in M_{\Omega }}y_{mn}>0\}}$$

(4) $$\sum_{m\in M_{\Omega }}y_{mn}\;=\;1$$

The constraint (3) represents the wavelength activation state, where $V_{n}=1$ represents that wavelength *n* is assigned to the RRU for data transmission. The constraint (4) imposes the wavelength availability constraint, i.e., ensuring that each RRU can only allocate a single wavelength. Since the clustering result of each transmission slot cannot be obtained by a closed expression, the problem is solved by reinforcement learning at this stage.

Assuming that BBU has stored a historical profile $\Gamma =\{\left\{H_{1},Q_{1}\right\},\left\{H_{2},Q_{2}\right\},\cdots$, $\left\{H_{T},Q_{T}\}\right\}$, the reinforcement learning agent obtains the wavelength allocation strategy by learning the historical environment. At the beginning of each transmission time slot, the agent selects a wavelength allocation strategy according to the ε−greedy and then executes CCDSA algorithms. The historical environment will feedback a reward signal $r_{a_{t}}$, which is the throughput that the current time slot unit fronthaul network can support at the current time slot, as shown in Equation (5).

(5) $$r_{a_{t}}=\frac{u(a_{t},H_{t},Q_{t})}{\sum_{n}V_{n}(a_{t})D_{\max}}$$

$u(a_{t},H_{t},X_{t})$ represents the revenue brought by the throughput of the current transmission time slot *t*, when action $a_{l}$, channel state information $H_{t}$ and request distribution $Q_{t}$ are determined. $\sum_{n}V_{n}(a_{t})D_{\max}$ shows the wavelength transmission cost of the system after a given action. According to the reward value of each time slot, the update criterion for determining the expected cumulative reward value is shown in Equation (6).

(6) $$Q_{a_{l}}^{t+1}=Q_{a_{l}}^{t}+\lambda_{t}\phi_{\left\{a_{t}=a_{l}\right\}}\left(r_{a_{t}}-Q_{a_{l}}^{t}\right)$$

Among them, $\phi_{\left\{a_{t}=a_{l}\right\}}$ indicates that the action is selected in the transmission time slot, and the optimal wavelength allocation result for each time frame can be expressed as $a^{*}=\underset{a_{l}}{\arg\max}Q_{a_{l}}$.

To sum up, we propose a RLWA algorithm as shown in Algorithm 1.
**Algorithm 1** Reinforcement Learning-based Wavelength Allocation Algorithm**Input:**   $\Gamma ,\varepsilon ,\lambda_{t}$**Output:**   Wavelength allocation matrix1:**Initialize Process:**2:Based on history network data $\Gamma =\{\left\{H_{1},Q_{1}\right\},\left\{H_{2},Q_{2}\right\},\cdots ,\left\{H_{T},Q_{T}\right\}\}$, the BBU initialize a virtual environment. Then a agent is created, whose action set is $A=\{a_{1},a_{2},\cdots ,a_{L}\}$. Initialize t=1, $Q_{a_{l}}=0$ for each $a_{l}$, the exploration probability ε and and learning rate $\lambda_{t}$.3:**Learning Process:**4:**for** each transmission interval t=1:T
**do**5:    Generate a random number *z*6:    **if**
z<ε
**then**7:        The agent selects a wavelength allocation strategy $a_{t}$ with equal probability.8:    **else**9:        The agent selects a wavelength allocation strategy with the maximum Q−value10:    **end if**11:    Under $a_{t}$, $H_{t}$ and $Q_{t}$, Algorithm 2 is performed to produce a clustering result12:    The environment feedbacks the signal $r_{a_{t}}$ to the agent13:    The agent makes an update according to Equation (5)14:**end for**

It is assumed that the channel distribution characteristic and request distribution characteristic remain unchanged for a long time. Then optimization problem (2)–(4) corresponding to the future period is the same as that of the historical period. In Algorithm 1, the historical information is used as the input to obtain the wavelength allocation strategy of the current time frame. In the beginning, the historical information Γ, exploration probability ε and learning rate $\lambda_{t}$ are initialized. In learning process, each agent selects an action and execute Algorithm 2. Finally, a certain wavelength allocation matrix *Y* is obtained according to the updated *Q* value.

The complexity analysis of Algorithm 1 is as follows. Since the complexity of generating a random number and *Q* value update is is O(1), the complexity of Algorithm 1 depends on the complexity of Algorithm 2, which is $O\left(M(K+M)RK\right)$.

### 4.3. CoMP Clustering and Dynamic Spectrum Allocation Algorithm

As the wavelength allocation results in a long timescale is determined, we propose a CCDSA algorithm in a short timescale, which includes the user cooperative set selection and spectrum allocation. In the short time scale, RRU wavelength assignment has been determined by the RLWA algorithm, the objective of CCDSA algorithm is to maximize the system throughput of each transmission slot.

Since the spectrum resources are limited, we consider a C-RAN based on OFDMA, in which the system bandwidth is divided into multiple orthogonal resource blocks. Let $R_{\Omega }\;=\;\left\{1,2,\cdots ,r,\cdots ,R\right\}$ denote the set of the resource block, which is the smallest unit of radio resource. To achieve users’ QoS demand, a user can be allocated multiple RBs. Let $z_{kr}$ denote the RB allocation indicator, whereas $z_{kr}\;=\;1$ means that RB *r* is allocated to the user *k*. Otherwise $z_{kr}\;=\;0$. In order to ensure the consistency constraint, we define $R_{k}=\left\{r|z_{kr}=1,\forall r\in R_{\Omega }\right\}$ as the RB set occupied by the user *k*. Considering the limitation of spectrum resources, assume that all RRUs share an RB resource pool and use RB resources in a certain proportion for data transmission.

To further improve the utilization of RB resources, the transmission mode is adaptive to the network environment, which is divided into a non-cooperative mode and CoMP-JT mode. In the non-cooperative mode, the user is served by only one RRU, while the cardinality of the cooperative set can be multiple in the CoMP-JT mode. Let $x_{mk}\;=\;1$ denote the user association indicator, whereas $x_{mk}\;=\;1$ means that RRU *m* participates in the data transmission service of the user *k*. Otherwise $x_{mk}\;=\;0$. Then the cooperative set of the user *k* can be defined as $S_{k}=\left\{m|x_{mk}\;=\;1,\forall m\in M_{\Omega }\right\}$, while the user set served by RRU *m* can be defined as $U_{m}=\left\{k|x_{mk}\;=\;1,\forall k\in K_{\Omega }\right\}$. Then the interference set of user *k* can be defined as $I_{k}=\left\{m|\exists r\in R_{\Omega },s.t.z_{kr}=z_{k^{\prime }r},\forall k^{\prime }\in U_{m}\right\}$, which refer to the RRUs use the same RB for transmission as the cooperative set of user *k*.

Assuming that the power allocated to each resource block is constant, the SINR of user *k* can be calculated as Equation (7).

(7) $$SINR_{k}=\frac{\sum_{m\in S_{k}}x_{km}Pl_{km}^{-\alpha }{\left|H_{km}\right|}^{2}}{\sigma^{2}+\sum_{m^{\prime }\in I_{k}}x_{km^{\prime }}Pl_{km}^{-\alpha }{\left|H_{km^{\prime }}\right|}^{2}}$$

When the user’s cooperative cluster and RB resources allocation are determined, the user’s achievable rate can be obtained as Equation (8).

(8) $$V_{k}=\sum_{r=1}^{R}z_{kr}w\log_{2}\left(1+SINR_{k}\right)$$

The user rate will be improved by adding more RRUs into the cooperative set. However, the fronthaul load will increase when RRU in the collaboration set does not cache the requested content. To exploit the broadcast characteristics of TWDM-PON, we can reduce the fronthaul load by promoting RRU cooperation at the same wavelength. For facilitate description, we define an auxiliary variable $\gamma_{nk}$, which represents the fronthaul load caused by the user *k*, shown in Equation (9).

(9) $$\gamma_{nk}={𝟙}_{\left\{\sum_{m\in M_{\Omega }}x_{mk}y_{mn}(1-c_{mf})>0\right\}}$$

Among them, $x_{mk}y_{mn}(1-c_{mf})=\;1$ indicates that the RRU *m* under the wavelength *n* serves the user *k*, and the user has to acquire the content by fronthaul network if there is no cache in the RRU of the cooperative set. When there is many RRUs in the cooperative set which satisfy $\sum_{m\in M_{\Omega }}x_{mk}y_{mn}\left(1-c_{mf}\right)>0$, then $\gamma_{nk}\;=\;1$. It is assumed that fronthaul bit rate $f_{k}$ is proportional to user rate, represented by $\eta V_{k}$, in which η is a constant parameter [29]. Then the clustering problem in each transmission slot can be modeled as follows.

(10) $$\max\sum_{k\in K}V_{k}\left(\psi_{Y}^{*}\left(H,Q\right)\right)$$

(11) $$\sum_{k\in K_{\Omega }}x_{mk}\sum_{r\in R_{\Omega }}z_{kr}\leq W$$

(12) $$\sum_{k\in K_{\Omega }}\sum_{r\in R_{\Omega }}x_{mk}z_{kr}=1,\forall m\in S_{k}$$

(13) $$\sum_{k\in K_{\Omega }}\gamma_{nk}f_{k}\leq D_{\max}$$

(14) $$V_{k}\geq V_{k}^{th}$$

The objective function (10) can be seen as the sum of the utility value seen by each user and the objective of proposed scheme is to maximize the sum rate of all users. Constraint (11) imposes the system bandwidth constraints, and constraint (12) represents the unique of radio frequency constraint for each user, i.e., ensuring that an RB should be allocated to only one user for an RRU. It is worth mentioning that RRUs under the same wavelength only transmits the user data once for cooperation, and the fronthaul load does not exceed the fronthaul capacity constraint, as shown in constraint (13). The constraint (14) is the QoS constraint, which means that the user rate should meet its minimum rate requirement. To improve user throughput, we select a cooperative set for each user under the constraints of radio resources and fronthaul resources. Since the association indicator and RB allocation indicator is highly coupled in Equation (12), the problem is NP-hard even if the number of RRUs and users are small.

More importantly, in a user-centric clustering process, the relationship between users and RRUs is many-to-many, making the problem harder to solve. Moreover, due to the radio frequency unique constraint, the optimization scheme should balance the gain of its signal quality and the loss of other user’s resource availability.

In this scenario, we propose a CCDSA algorithm based on overlapping coalition formation (OCF) game, in which players can belong to multiple coalitions and contribute their resources among these coalitions. It is assumed that RRUs are the players that join any coalition. Here, each coalition basically represents one cooperative set providing service for a user. Therefore, each coalition will have one user and one or more RRUs. Each user contributes RBs to the coalition it joins.

**Definition** **1.** *OCF game for CoMP clustering: The proposed overlapping coalition game G(B,v,F) is a composite function of an RRU set. RRU can join any coalition and form a coalition structure with a coalition set $F=\{F_{1},F_{2},\cdots ,F_{k}\}$ and a utility function $v:{\left[0,1\right]}^{K}\to R^{+}$, in which $F_{k}$ represents the RRUs serve the user k.*

In an OCF game, we need to redefine the utility of each member in a coalition for decision. In a user-centric cluster, the user rate is jointly determined by all RRUs in the cooperative set, and the sum-rate cannot represent the utility of RRU. Then we define a gain factor, which indicates the importance of RRU to user QoS improvement, as shown in Equation (15).

(15) $$\beta_{m}^{k}=\frac{\gamma_{k}\left(F_{k}\right)-\gamma_{k}\left(F_{k}\backslash \{B_{m}\}\right)}{\gamma_{k}\left(F_{k}\right)}$$

$\gamma_{k}\left(F_{k}\backslash \{B_{m}\}\right)$ represents the SINR of the user *k* can reach after removing from user’s cooperative set $B_{m}$. For the same RB allocation, RRU with higher SINR can raise user’s rate gain. Since the wavelength allocation is determined in the short timescale, the fronthaul efficiency is also higher. Then the contribution of each RRU to the coalition can be expressed as Equation (16).

(16) $$\eta_{m}^{k}=\frac{\beta_{m}^{k}}{\sum_{m^{\prime }\in F_{k}}\beta_{m^{\prime }}^{k}}$$

Let $F_{m}^{*}$ denoote the coalition set that RRU $B_{m}$ belongs to, which also represents the user set of $B_{m}$. The individual utility of the member $B_{m}$ of the coalition $F_{m}^{*}$ can be calculated according to the weighted sum rate of users served by $B_{m}$, in which $V_{k}$ is calculated in Equation (8).

(17) $$v\left(B_{m}^{F_{m}^{*}}\right)=\sum_{k\in F_{m}^{*}}\eta_{m}^{k}V_{k}$$

Let $v(F_{k})$ denote the utility of the coalition $F_{k}$, which is also the rate of the user *k*, as shown in Equation (18).

(18) $$v\left(F_{k}\right)=V_{k}^{F_{k}}$$

Then we can represent the total utility of a coalition structure *F* as the sum of the utility of all coalitions in the structure as Equation (19).

(19) $$v\left(F\right)=\sum_{k\in F_{k}}R_{k}^{F_{k}}=\sum_{m\in M_{\Omega }}\sum_{k\in F_{m}^{*}}\eta_{m}^{k}V_{k}$$

In the game process, RRU joins a coalition $F_{k}$ to increase the coalition’s utility by improving the rate of the user *k*. However, when the RRU load is high, the newly added user will reduce the rate of other users under the same RRU. Meanwhile, the utility of coalitions that occupy the same RB will also decrease due to co-channel interference. Considering the utility of both individual and coalition, we define the switching rules to select RRU to form the coalition $F_{k}$, as shown in Definition 2.

**Definition** **2.** *Switching rule: Consider a coalition structure $F^{\prime }=\left\{F_{1},\cdots ,F_{h},\cdots F_{g},\cdots ,F_{k}\right\}$, if $B_{m}$ wants to be independent of $F_{h}$ and join $F_{g}$, a new coalition structure $F^{\prime \prime }=\{F_{1},\cdots ,F_{h}^{q},\cdots F_{g}^{q},$$\cdots ,F_{k}\}$ is formed, where $F_{h}^{q}=F_{h}\setminus \left\{B_{m}\right\}$ and $F_{g}^{q}=F_{g}\cup \left\{B_{m}\right\}$. The process above can be described as a switching rule ${≻}_{m}$, which represents that $B_{m}$ prefers being a member of $F^{\prime }$ than that of $F^{\prime \prime }$.*

When an RRU $B_{m}$ wants to form a new structure, the switching condition must be satisfied: (i) the utility of RRU $B_{m}$ is improved. (ii) the rate of the user *k* is improved. (iii) the value of the new structure is increased. (vi) the wavelength capacity constraint is satisfied, as shown in Equation (20).

(20) $$F^{\prime }{≻}_{m}F^{\prime \prime }\Leftrightarrow \left\{\begin{array}{c} v\left(B_{m}^{F_{m}^{*}},F^{\prime }\right)>v\left(B_{m}^{F_{m}^{*}},F^{\prime \prime }\right)\\ v\left(F_{h}^{q},F^{\prime }\right)>v\left(F_{h},F^{\prime \prime }\right)\\ v\left(F^{\prime }\right)>v\left(F^{\prime \prime }\right)\\ \sum_{B_{m}\in B}\gamma_{nk}\left(1-c_{mf}\right)fh_{k}\leq D_{\max} \end{array}\right.$$

**Figure 3:**
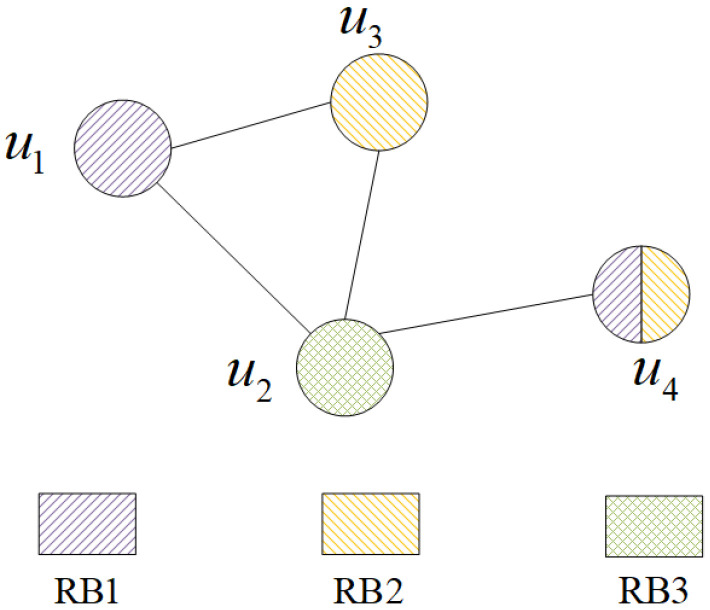
Dynamic Spectrum Allocation.

Note that the increase in RRU load leads to increasing interference among conflicting users who are allocated the same RB. To address this issue, we optimize the RB allocation when the coalition structure is changed. Since the cooperative set is overlapped, the traditional resource allocation algorithms for FDMA cannot be directly applied to this scenario. Then we adopted a list coloring algorithm to allocate RB for each cooperative set [32]. In the proposed algorithm, the interference of overlapping cooperation cluster can be modeled using a graph I(U,E), as illustrated in Figure 3.

In *I*, $U=\{u_{1},u_{2},\cdots \}$ is the vertex of the graph *I*, which represents the set of users. If the cooperate set of $u_{a}$ and $u_{b}$ have common RRUs, we set $E(u_{a},u_{b})=1$. The algorithm minimizes the total number *J* used in the graph, which represents that the system band is divided into *J* equal-bandwidth subbands. The bandwidth allocated to the user *k* is $W_{k}=r_{k}W/J$, where $r_{k}$ indicates the proportion of bandwidth occupied by the user *k*.

In summary, we propose a CCDSA algorithm as shown in Algorithm 2. In order to avoid an RRU repeatedly joining the same coalition in the process of coalition structure update, a historical profile $H(B_{m})$ is introduced to record the coalitions that the RRU has joined. CCDSA algorithm contains a two-stage process, including the coalition initialization process and the coalition update process. To minimize the fronthaul load, cache based cluster formation is performed in the initialization process. Each user selects the neighboring RRU with the requested content as the cooperative set. The neighboring RRU of a user means the RRU user can associate with maximum SINR. In the update process, algorithm performs iterative leave and join operations. In leave operation, for users who do not meet the rate requirements, the RRU with the smallest contribution is removed from their cooperative set. Then the algorithm traverses other members in the coalition, and users whose rate cannot meet the requirements are temporarily dropped from the user set of the member. It is based on the rationale that user’s cooperative set may be suboptimal because of the weak user selecting a weak link or insufficient RB resource allocated by the RRU in the cooperative set. Then RRUs have the opportunity to form a new coalition structure with better performance. In join operation, The user chooses new RRUs to join the cooperative set according to the switching rule. To further improve the system throughput, each RRU traverses coalitions not in $H(B_{m})$ and updates the coalition structure repeatedly until the structure is stable.

The properties of Algorithm 2 are as follows.

Convergence: In a given network architecture, the number of users, RRUs, and available RBs in the network are all fixed. Therefore, the number of all possible coalition structure is also fixed. In the proposed algorithm, each RRU switches to the new coalition with higher system throughput than the primary structure, and the proposed algorithm avoids the RRU joining the same coalition twice. Therefore, our solution can converge to a stable overlapping coalition structure.

Stability: According to the algorithm, the final coalition structure $F^{\prime }$ generated is stable. Assume that the final coalition structure $F^{\prime }$ is unstable, and there exists RRU *m* to be resigned to improve system throughput. The RRU reassignment would generate a new coalition structure $F^{\prime \prime }$. Therefore, $F^{\prime }$ is not the final coalition structure generated by the convergence of the proposed algorithm, then the assumption does not hold. From the above, the final coalition $F^{\prime }$ is stable.

Complexity: The complexity of Algorithm 2 is highly related to the channel distribution and fronthaul load generated by the network environment. Then we consider its worst-case execution as follows. In the coalition structure update process, the leave and join operation checks up to KM switching rules. Then the traverse operation takes most $M^{2}$ time. Besides, the spectrum optimization complexity is solved through the list coloring algorithm, which takes $O\left(RK\right)$ time to allocate the spectrum for each user [32]. Therefore, the overall complexity is $O\left(M(K+M)RK\right)$.
**Algorithm 2** CoMP Clustering and Dynamic Spectrum Allocation Algorithm**Input:**   $K_{\Omega },M_{\Omega },H,Q,C,Y$**Output:**   Clustering Result and RB Allocation;1:**Initialization:**2:Each user selects the neighboring RRU with the requested content as the cooperative set3:**Coalition Structure Update Process:**4:**for**$k\in K_{\Omega }$**do**5:    **while**
$V_{k}<V_{k}^{th}$
**do**6:        **for**
$B_{m}\in F_{k}$
**do**7:            **if**
$\eta_{m}^{k}V_{k}$ is minimum of all $\eta_{m^{\prime }}^{k}V_{k},\forall B_{m}\in F_{k}$
**then**8:                $F_{k}\leftarrow F_{k}-\left\{B_{m}\right\}$9:            **else if**
$V_{k^{\prime }}^{F_{m}^{*}}<V_{k^{\prime }}^{th}$
**then**10:                $F_{m}^{*}\leftarrow F_{m}^{*}-\left\{k\right\}$ and update RB Allocation11:            **end if**12:        **end for**13:        **for**
$m^{\prime }\in \left\{M_{\Omega }-F_{k}\right\}$
**do**14:            **if**
$v\left(F_{k}\cup m^{\prime }\right)>v\left(F_{k}\right)$
**then**15:                $v\left(B_{m^{\prime }}^{F_{m^{\prime }}^{*}\cup k}\right)>v\left(B_{m^{\prime }}^{F_{m^{\prime }}^{*}}\right)$ and (13) satisfied16:                $F_{m^{\prime }}^{*}\leftarrow F_{m^{\prime }}^{*}\cup \left\{k\right\}$ and update RB Allocation17:            **end if**18:        **end for**19:    **end while**20:**end for**21:**Repeat:**22:**for**$m\in M_{\Omega }$**do**23:    update $F_{m}^{*}$ according to formula (20) and $H(B_{m})$, update RB Allocation24:**end for**25:**Until** stable coalition structure

## 5. Simulation Results and Analysis

### 5.1. Comparison Algorithm and Simulation Setup

In this section, we analyze and verify the proposed scheme in a Python simulation environment, and the convergence and efficiency of the proposed algorithm are proved. The simulation is carried out in an area of 500 m × 500 m, and the locations of RRU and user are randomly generated by Poisson distribution. The channel gain is composed of small scale fading and path loss. The small scale fading follows a complex Gaussian distribution with a mean of 0 and a variance of 1. Refer to [33], the transmit power of RRU is set to −40 dBm, the spectral density of white noise is −169 dBm, and the path loss index is 4. At each time slot, the channel matrix is generated randomly. Besides, it is assumed that the file library contains 100 contents and user randomly produces a request according to the Zipf distribution. The requesting probability for content is shown in Equation (21). A TWDM-PON fronthaul network with four wavelengths is considered. The capacity of each wavelength is 2.5Gbps, and η is set to 100 [34]. The simulation parameters are shown in Table 1.

(21) $$P_{f}=\frac{1/f^{\gamma }}{\sum_{q=1}^{F}1/q^{\gamma }}$$

To evaluate the performance of the proposed scheme, we compare our proposed MRSCT scheme with the existing works from the wireless and optical domains. The comparison algorithms in the wireless domain include the non-cooperative (NOC) algorithm, the joint user-centric overlapped clustering and resource allocation (JCOCRA) algorithm proposed in [20], and the network-centric clustering (NCC) algorithm proposed in [31]. Among them, NOC is the baseline algorithm, in which users associate with RRU based on maximum SINR, without cooperation among RRUs. The NCC is a network-centric clustering algorithm in which RRUs form disjoint coalitions. The JCOCRA designs coverage distance-based user-centric overlapped clustering solution through a two-sided matching algorithm, in which the interference-aware spectrum allocation algorithm is included. In the optical domain, the performance of the wavelength allocation algorithm based on reinforcement learning is verified by comparing with the fixed wavelength allocation algorithm.

### 5.2. Performance Evalution

**Figure 4:**
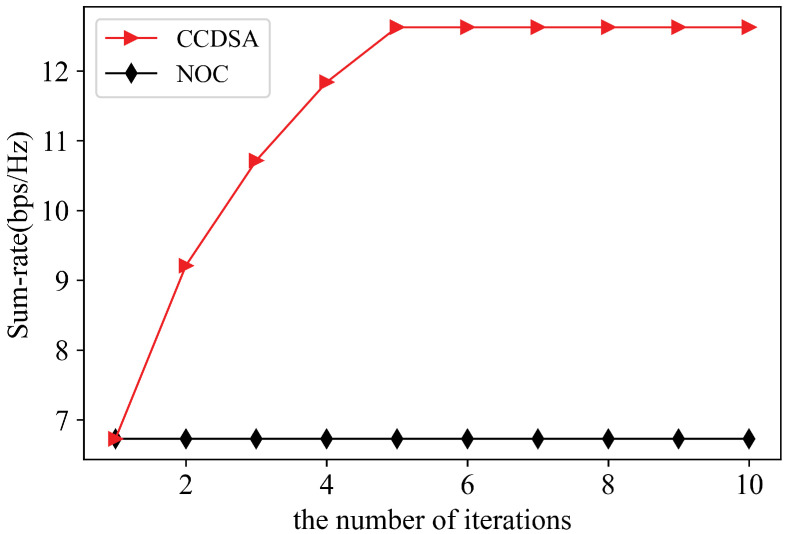
Convergence Analysis of CoMP clustering and dynamic spectrum allocation (CCDSA).

We first verify the convergence of CCDSA in the scenario where the location of users, request matrix, and channel gain matrix are all randomly generated under γ=0.5. Then we create a network with 7 RRUs generated by following poison distribution. Users are generated similarly. The ratio of user density to RRU density is set to 10. The RRU randomly caches the 10 files among the most popular files. RRU randomly caches the files among the most popular files. In Figure 4, it is especially noted that CCDSA converges within only five iterations, which means that the algorithm can well adapt to the dynamic environment. Moreover, the performance of CCDSA is nearly doubled compared to the case of no cooperation.

**Figure 5:**
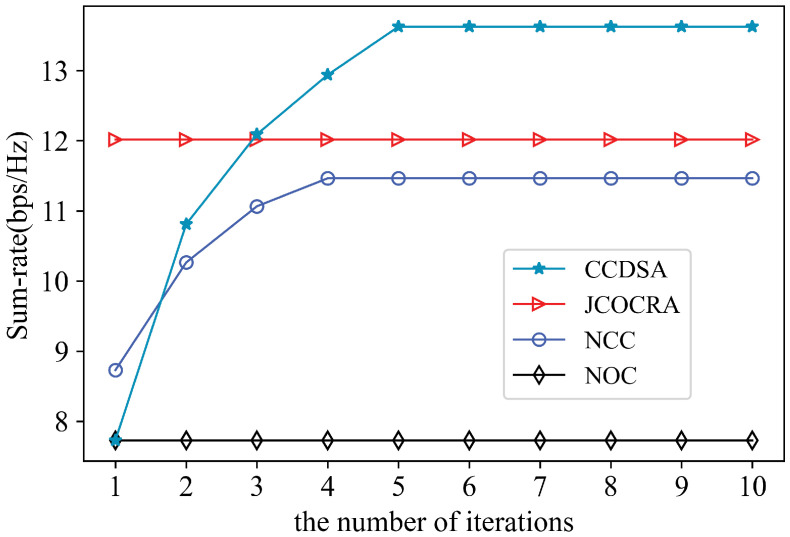
Sum-rate comprison ofdifferent schemes.

Figure 5 shows theperformance comparison of different algorithms. In the initial iteration of the algorithm, the sum-rate of the NCC and JCOCRA algorithms is higher. Because the proposed algorithm uses the cache-aware association decision at the beginning, which cannot ensure the best channel quality. However, as the number of iterations increases, the proposed algorithm can further increase the user’s rate through coalition structure update under the limitation of fronthaul capacity.

**Figure 6:**
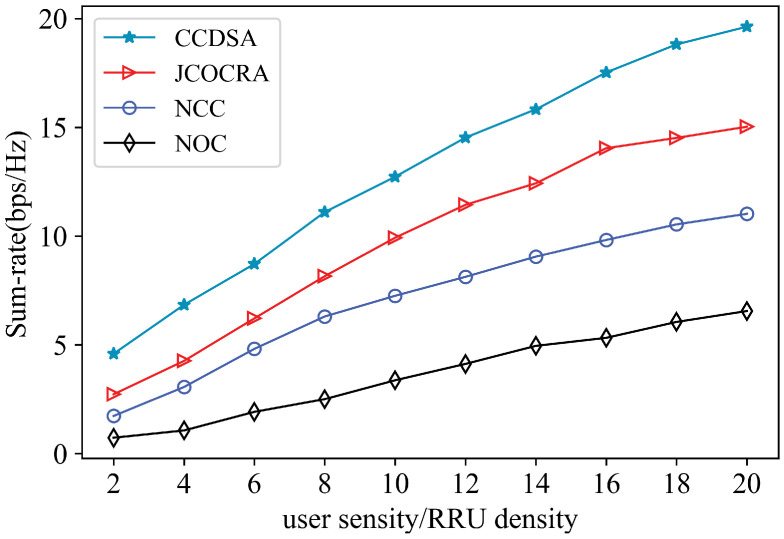
Sum-rate comparison of different scheme under different user density.

Figure 6 illustrates the sum-rate performance of CCDSA algorithm over other algorithms with different network loads. Observe that, when user density is low, the gap among four algorithms is small since the spectrum resource and fronthaul bandwidth is sufficient for user services. As the user density increases, the sum-rate improves. However, the performance improvement of NCC is limited since the network-centric clustering cannot avoid inter-cluster interference. In all cases, CCDSA algorithm always performs the best; meanwhile, when user density is high, CCDSA algorithm can achieve greater performance gains under the limitation of fronthaul capacity and spectrum resources.

**Figure 7:**
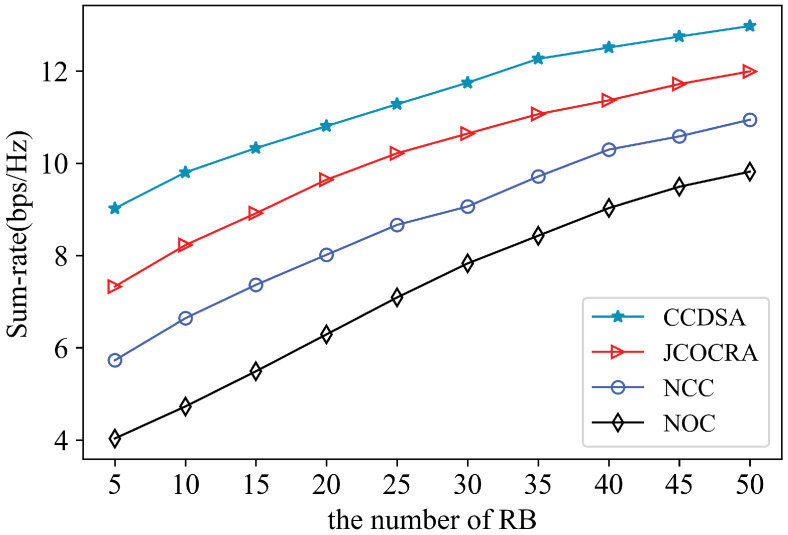
Sum-rate comparison of different scheme under different numbers of RB.

The performance is further investigated here as a function of the number of RBs in the network. It is clearly observed in Figure 7 that the sum-rate is increased with the number of RBs. This implies that more RBs lead to having more cooperating RRUs ready to serve more users. If the number of RBs is sufficiently high for all users, the gap among different schemes is small; otherwise, our scheme shows better performances, because our scheme can balance the load among RRUs and make full use of RBs through the joint design of the user’s cooperation set selection and resource allocation.

To obtain a wavelength allocation strategy through RLWA algorithm, a historical environment composed of 10,000 randomly generated tuples {H,Q} is created. To facilitate comparison, additional 1000 time slots are randomly generated to simulate the future period. At the beginning of the future period, the wavelength allocation matrix under different algorithms is initialized and remains unchanged in the subsequent 1000 time slots. Then fronthaul efficiency can be calculated by the average system throughput.

**Figure 8:**
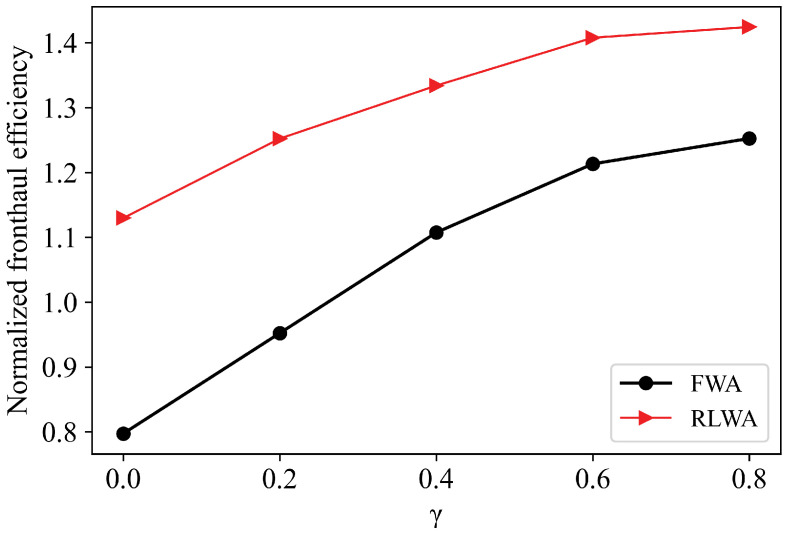
Impact of γ on fronthaul efficiency.

In Figure 8, we compare the sum-rate performance of different wavelength allocation schemes with different Zipf parameters γ. Assuming that each user randomly generates a request in the transmission slot, with each RRU storing 10 files. It can be seen that, as γ increases, users concentrate more on fewer contents, and the sumrate significantly improves. Moreover, the performance gaps between the proposed algorithm and FWA become smaller since cache reduces RRU’s demand for fronthaul bandwidth. When γ is small, the proposed algorithm promotes RRU cooperation opportunities through wave-length reconstruction, which can bring higher system gain under the dispersion of content popularity distribution.

**Figure 9:**
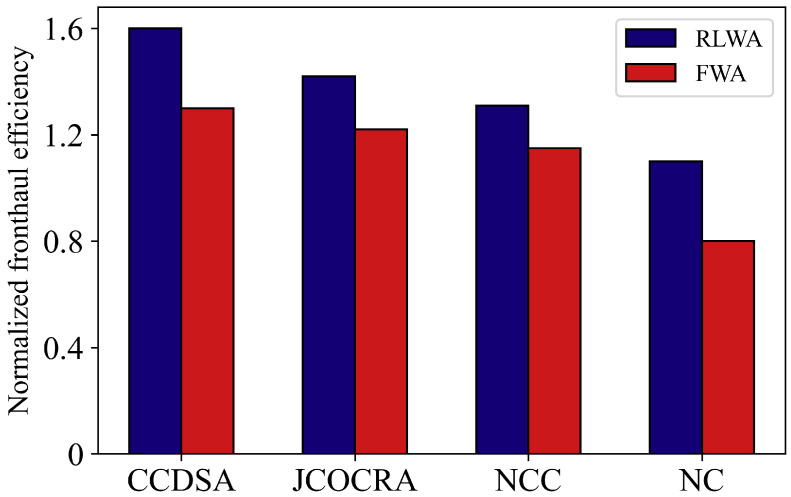
Comparison of fronthaul efficiency of different schemes.

In Figure 9, we compare the fronthaul efficiency of different schemes. Compared FWA, RLWA algorithm performs better over each clustering scheme. Because the intelligent agent learns the network environment in a long time scale and adjusts the wavelength selection of each RRU according to the best clustering scheme so as to improve the long-term effectiveness of the system. On the other hand, the clustering scheme in a short time scale will also greatly impact system performance. It can be seen from Equation (1), since the fronthaul load is fixed given wavelength allocation, the improvement of user rate caused by clustering strategy in each time slot can further raise the system fronthaul efficiency gain.

## 6. Conclusions

In this paper, we propose a user-centric cooperative transmission scheme in C-RAN based on TWDM-PON and designed a multi-dimensional resource schedule considering both wireless resource and wireless resource constraints. To solve this problem, we propose a two-timescale resource allocation framework. In a long timescale, the fronthaul network is reconstructed with the goal of maximizing the system’s long-term fronthaul efficiency, considering the fronthaul load. In a short timescale, the cooperative cluster selection of users and dynamic spectrum resource allocation are combined to improve system throughput, in which time-domain transmission resources are allocated through clustering based on overlapping coalition formation games. The simulation shows the effectiveness of the proposed clustering algorithm and the superiority of the wavelength resource allocation algorithm based on reinforcement learning.

In our current work, we adopt the random caching scheme, without considering the caching optimization. In practice, the placement of content will have a great impact on the fronthaul load and affect the cooperation gain. The joint design of the fronthaul bandwidth allocation and caching scheme is an interesting direction to explore in the future, and compromise of the fronthaul bandwidth and storage space of RRU should be considered. 

## Figures and Tables

**Table 1:** Simulation parameters.

Simulation Parameter	Value
Simulation area size	500 m × 500 m
Bandwidth of each resource block	150 kHz
Channel fading	complex Gaussian, 0 mean, 1 variance
Transmit power	−40 dBm
White noise power spectral density	−169 dBm
Content Library size	100
Content popularity	Zipf distribution
Exploration probability	0.9
Learning rate	0.5
Wavelength capacity	2.5 Gbps
Ratio of fronthaul load to traffic	100

## Data Availability

Data sharing not applicable.
